# Molecular phylogenetics reveal multiple tertiary vicariance origins of the African rain forest trees

**DOI:** 10.1186/1741-7007-6-54

**Published:** 2008-12-16

**Authors:** Thomas LP Couvreur, Lars W Chatrou, Marc SM Sosef, James E Richardson

**Affiliations:** 1 Nationaal Herbarium Nederland, Wageningen branch, Biosystematics Group, Wageningen University, 6703 BL, Wageningen, The Netherlands; 2 Royal Botanic Garden Edinburgh, Edinburgh, EH3 5LR, UK; 3 The New York Botanical Garden, Bronx, NY, 10458-5126, USA

## Abstract

**Background:**

Tropical rain forests are the most diverse terrestrial ecosystems on the planet. How this diversity evolved remains largely unexplained. In Africa, rain forests are situated in two geographically isolated regions: the West-Central Guineo-Congolian region and the coastal and montane regions of East Africa. These regions have strong floristic affinities with each other, suggesting a former connection via an Eocene pan-African rain forest. High levels of endemism observed in both regions have been hypothesized to be the result of either 1) a single break-up followed by a long isolation or 2) multiple fragmentation and reconnection since the Oligocene. To test these hypotheses the evolutionary history of endemic taxa within a rain forest restricted African lineage of the plant family Annonaceae was studied. Molecular phylogenies and divergence dates were estimated using a Bayesian relaxed uncorrelated molecular clock assumption accounting for both calibration and phylogenetic uncertainties.

**Results:**

Our results provide strong evidence that East African endemic lineages of Annonaceae have multiple origins dated to significantly different times spanning the Oligocene and Miocene epochs. Moreover, these successive origins (c. 33, 16 and 8 million years – Myr) coincide with known periods of aridification and geological activity in Africa that would have recurrently isolated the Guineo-Congolian rain forest from the East African one. All East African taxa were found to have diversified prior to Pleistocene times.

**Conclusion:**

Molecular phylogenetic dating analyses of this large pan-African clade of Annonaceae unravels an interesting pattern of diversification for rain forest restricted trees co-occurring in West/Central and East African rain forests. Our results suggest that repeated reconnections between the West/Central and East African rain forest blocks allowed for biotic exchange while the break-ups induced speciation via vicariance, enhancing the levels of endemicity. These results provide an explanation for present day distribution patterns and origins of endemicity for African rain forest trees. Moreover, given the pre-Pleistocene origins of all the studied endemic East African genera and species, these results also offer important insights for setting conservation priorities in these highly diversified but threatened ecosystems.

## Background

**Figure 1:**
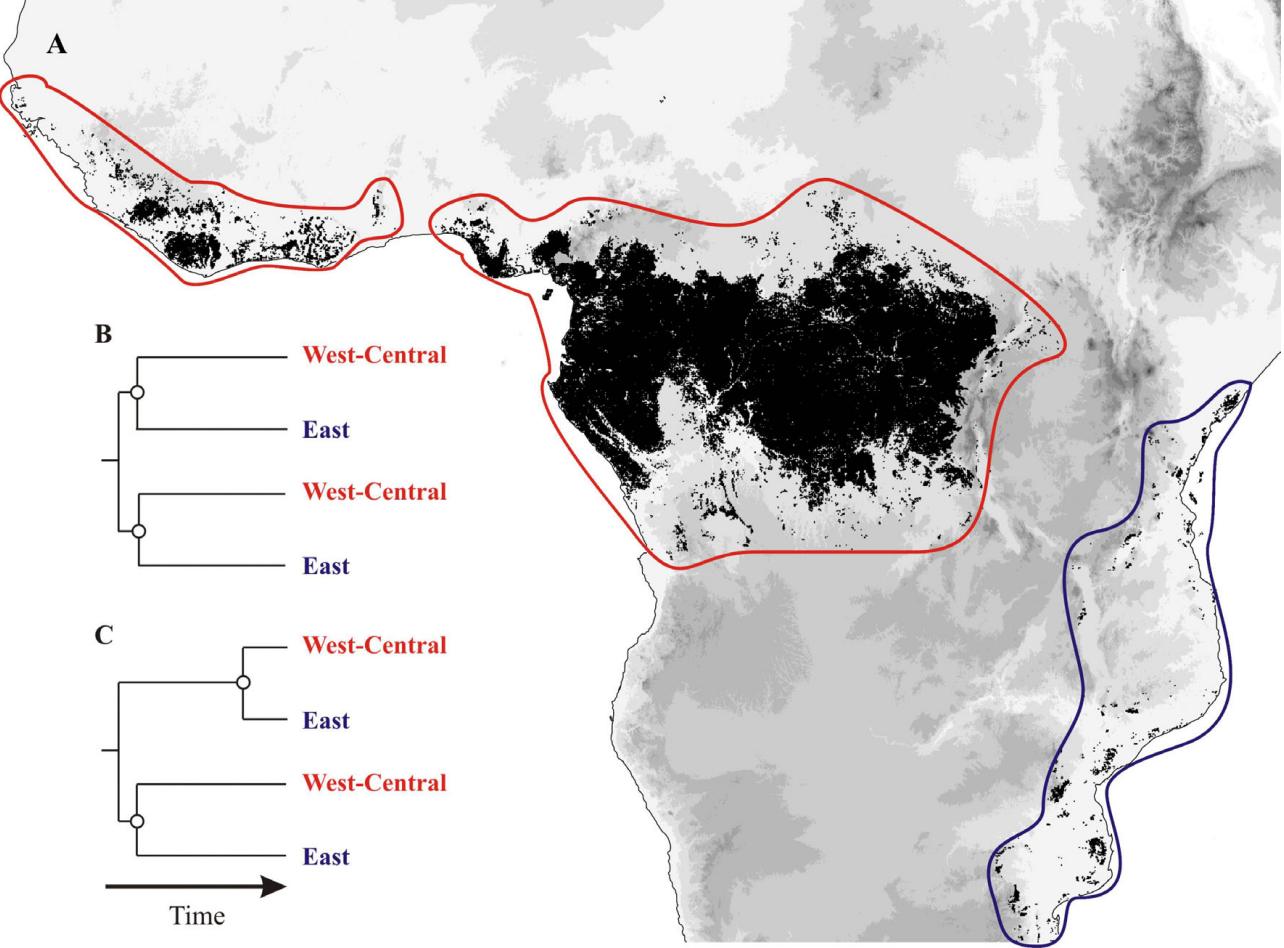
**Alternative hypotheses of African rain forest origins**. (A) Distribution of lowland rain forest in Africa (black) overlaid by altitudinal range (increasing altitude with darker grey). Red lines highlight the Guineo-Congolian region; the blue line highlights the East African region. (B) Phylogenetic tree expected from a single break-up scenario. (C) Phylogenetic tree expected from multiple break-ups at significantly different times scenario. Open circles indicate West-Central/East splits.

Tropical rain forests harbor outstanding levels of biodiversity. Numerous studies have focused on how such high levels of species richness might be maintained through time [1-3]. Much less is known about the evolutionary origins that have led to the generation of these hyper-rich ecosystems [4,5]. Understanding the evolutionary history and biogeographic processes behind the origin of such areas plays a central role in setting conservation priorities [6,7]. In Africa, rain forests of the Guineo-Congolian floristic region [8] occur in a coastal band in West Africa and occupy vast areas in Central Africa (Figure 1A). In East Africa, rain forests occur in small, highly fragmented patches near the coast and in the adjacent Eastern Arc Mountains (Figure 1A), covering a small surface area (c. 7000 km^2^). Despite the relatively small size of the area, the East African rain forests harbor an exceptional density of endemic plant species [9,10], which is one of the highest on the planet [7], and have been identified as one of 25 global biodiversity hotspots [7]. The Guineo-Congolian and East African regions are geographically isolated by a c. 1000 km-wide North-South arid corridor creating an effective barrier to dispersal for rain forest-restricted taxa. Nevertheless, floristic comparisons indicate strong affinities between the two rain forest regions within numerous distantly related plant families [8,9,11,12]. Indeed, many endemic species are representatives of widespread genera co-occurring in the Guineo-Congolian and East African rain forest regions. In addition, the endemic East African genera are thought to be closely related to Guineo-Congolian genera. These strong affinities are generally explained by the presence of a continuous rain forest across tropical Africa during the Eocene [9-16].

One hypothesis for explaining the high levels of endemicity suggests that this pan-African rain forest broke up only once at the onset of East African aridification during the Oligocene-Early Miocene (c. 33-20 Myr) [11-13,15,17] and that the c. 30 Myr of isolation of the East African rain forests coupled with a highly fragmented habitat led to the high levels of endemicity observed in East Africa. In contrast, an alternative hypothesis suggests that from the mid-Tertiary onwards (c. 33-2 Myr) African rain forests expanded and contracted on multiple occasions after the initial break-up [9,12,14,16]. Expansion and reconnection would have allowed new lineages to spread from West-Central Africa to East Africa or vice versa. Subsequent contraction and isolation would have resulted in diversification by vicariance or allopatric speciation, thus enhancing the levels of biodiversity and endemicity.

In the context of studying the origins of East African dry-adapted endemic species in the genus *Acridocarpus *(Malpighiaceae), dated molecular phylogenies have proven to be a powerful tool [17]. Here we use a similar approach on a rain forest-restricted clade with disjunct West-Central and East African distributions, which provides a way to test the above hypotheses. If a single break-up of the pan-African rain forest is responsible for the observed distribution, then all the splits between Guineo-Congolian and East African lineages should be dated to the same period (Figure 1B). In contrast, in an oscillating forest scenario we expect to find a chronological sequence of multiple vicariance events, with splits dated at significantly different time periods (Figure 1C).

We studied the evolutionary history of the largest monophyletic African clade of rain forest trees [18] within the diverse pan-tropical family Annonaceae. Annonaceae is one of the best examples of a tropical plant family for which strong positive correlations were found between abundance and species richness, and temperature and rainfall, respectively [see [19]]). This implies that Annonaceae are mainly restricted to tropical rain forests and thus provide an ideal model family to test hypotheses on the evolution of rain forests. The studied African clade is composed of three genera endemic to the East African rain forests, two endemic to the Guineo-Congolian region, and six disjunct genera with numerous species endemic to either East or West-Central African rain forests [20]. These genera are not found in arid parts of Africa and are restricted to forests with high rainfall regimes and relatively short dry seasons. Moreover, the comparatively large fruits found within this group are not adapted to long-distance dispersal. Within this clade, monocarps are sessile in genera with apocarpous fruits [18] and differ from the stalked monocarps that are more common in Annonaceae. So, even though individual fruitlets are free, in the context of dispersal these genera have fruits that functionally are equivalent to syncarpous fruits, far too large for effective long-distance dispersers such as birds. Rather, they are dispersed by mammals that are restricted to rain forests, such as gorillas [21], and their crossing wide arid corridors is a highly improbable event. This low dispersal capacity as well as the restriction and abundance of this clade in both the Guineo-Congolian and East African rain forests [11,20,22] renders this clade suitable for testing the above hypotheses.

Two dated, well-resolved molecular phylogenies were generated using plastid DNA sequences, focusing specifically on two different taxonomic levels within the African clade. The first phylogeny contained representatives of all eleven African genera and was used to date the deeper splits within this clade. Second, a species-level phylogeny was estimated for two sister genera, *Isolona *and *Monodora*, each of which has endemics in both Guineo-Congolian and East African rain forests [20,23]. Both trees were dated under a Bayesian relaxed uncorrelated molecular clock assumption.

## Methods

### Taxon sampling and sequence data

For this study two different chloroplast DNA sequence datasets were used. First, in order to date the origin of the East African endemic genera, a previously published DNA sequence data matrix (dataset A in Additional file 1) of the African clade was used (Couvreur et al., 2008, treeBASE SN3554). This dataset, totaling 64 taxa, included all the genera of the African clade (11 genera) and presented a thorough sampling of the genera within the long branch clade [24]. The family Eupomatiaceae was used as the outgroup [Eupomatiaceae has been recovered as sister to Annonaceae, [25,26]]). The dataset was composed of six plastid markers, three non-coding (*trnL-trnF*, *trnSG*, and *psbA*-*trnH*) and three coding (*ndhF*, *rbcL*, and partial *matK*), totaling 7945 characters.

Second, in order to date the splits within genera, a species-level phylogeny of the two sister genera *Isolona *(15 out of 20 species) and *Monodora *(13 out of 14 species) was generated (dataset B, see Additional file 1, treeBASE number SN3633). For both genera all the known East African species were included.

A modified cetyl trimethyl ammonium bromide (CTAB) protocol of Doyle and Doyle [27] following Bakker et al. [28] was used for DNA extraction. The universal primers C/D and E/F [29] were used to amplify and sequence the *trnL *intron and *trnL-trnF *spacer. The *psbA-trnH *intergenic spacer was amplified and sequenced using primers *psbA *and *trnH *(GUG) [30]. The *trnS-trnG *intergenic spacer was amplified and sequenced using primers trnS (GCU) and trnG (UCC) [30] and the *trnD*-*trnT *marker was also amplified and sequenced using primers trnT^GGU ^and trnD^GUC ^[31]. In addition, the second part of the *ndhF *gene was sequenced as it has been shown to be more variable than the more conserved 5' region and is thus more appropriate for species-level analyses [32]. The Annonaceae specific primer LBC-intF [32] was used in combination with the usual 2110R primer [33] amplifying a region of c. 620 bp. PCR reactions were performed with 30–50 ng of genomic DNA, 0.4% of BSA, 0.2 μM of each primer, 0.2 mM dNTP PCR mix (Promega, Madison, WI), 3 μM MgCl_2_, 1× PCR buffer (Promega, Madison, WI), and 0.5 U of *Taq *DNApolymerase (Promega, Madison, WI) in a total volume of 50 μl. The PCR program was as follows: 35 thermal cycles at 94°C for 1 minute, 50–55°C for 50 seconds, 72°C for 50 seconds and a final extension at 72°C for 3 minutes.

For both datasets, sequences were edited using the program Staden [34] and aligned manually using PAUP* [version 4.10b; [34]]. Gaps were coded following the simple coding model of Simmons and Ochoterena [35]. Microsatellites were excluded from the analysis, as these structures probably originate through slipped-strand mispairing [36] and are likely to be highly homoplasious.

### Phylogenetic analysis and divergence date estimates

In order to test whether the estimated dates of the origin of the East African endemic lineages are significantly different the molecular trees were dated under a Bayesian framework using the software BEAST v. 1.4.7 [37,38]. The method implemented in BEAST simultaneously estimates divergence times, tree topology, and rates, thereby providing a clear advantage over previous relaxed clock methods [39] that estimate tree topology and divergence dates separately [e.g. [40-42]]). Both datasets were partitioned into the number of markers used by directly editing of the XLM file and following Couvreur [44]. The best performing evolutionary model for each marker was identified under two different model selection criteria, the hierarchical likelihood ratio test (hLRT) and the Akaike information criterion [AIC; [43]]) as implemented in MrModelTest [44]. For both datasets two independent analyses were undertaken to check for convergence of the MCMC chains. Analyses were undertaken by sampling every 1000th generation, and were considered complete once the effective sampling size (ESS) of each parameter was above 200, as suggested in the BEAST manual. In order to assess that the MCMC chain reached stationarity we examined the lnL plots using Tracer v. 1.3 [45]. In particular, we searched for evidence that the model likelihood and parameter estimates had reached stationarity after a burn-in period.

### Calibration

The testing of the two alternative hypotheses presented in Figure 1 can be viewed under a relative time scale [46], i.e. with no reference to any absolute timing. However, fossil information is available and was used in order to calibrate trees in an absolute time frame. The fossil taxon *Archaeanthus *[98 Myr, [47]]) is characterized by distinctive derived stipules, an elongated receptacle, and fruits with numerous well spaced follicles, all of which are interpreted as synapomorphies shared with the family Magnoliaceae [47-49]. Thus, this fossil provides a minimum age for the stem of Magnoliaceae of 98 Myr. Even though the correct placement of this fossil within the Magnoliales has been questioned in the past [50], recent studies [24,48,51] have largely relied on *Archaeanthus *to date this family. Fossil information within Annonaceae is limited ([e.g. [52,53]]) and is thought [24,48] to be unreliable with respect to their exact placement and was thus not used in this study. One other relevant fossil is the Early Cretaceous *Endressinia *taxon (112 Myr [56]). This fossil is characterized by broad staminodes with lateral glands, which implies a possible sister relationship to Eupomatiaceae and would thus provide an older minimum age of 112 Myr for the split between Eupomatiaceae and Annonaceae than with *Archaeanthus*. However, the method used to assign *Endressinia *to Eupomatiaceae (see [57]) has been criticized and is thought to be unreliable [58]. Thus, we deemed *Archaeanthus *the most reliable fossil calibration point and this was used to assign a minimum age to the Magnoliaceae stem node.

Using the age of *Archaeanthus *as well as a wider taxon sampling within Annonaceae (80 genera out of c. 110, 205 OTUs) and *rbcL *and *trnLF *sequences, Pirie [59] provided an age estimate of 91 Myr (± 1.5 Myr) for the stem of Annonaceae (split between Eupomatiaceae and Annonaceae) when using the Penalized Likelihood method of Sanderson [43]. This estimated date is in accordance with previously published date estimates for the origin of Annonaceae [24,60]. Thus, 91 Myr was used as a calibration point for dataset A. Secondary calibration (calibrating a node with a date provided by a previous analysis) is a commonly used alternative given the absence of a method for direct calibration (e.g. fossil or geological [61-63]). However, it has been shown that, unless particular care is taken, secondary calibration can generate internal inconsistencies leading to unreliable dates [64]. BEAST accommodates for calibration uncertainty by applying a prior probability distribution on the age, e.g. a prior distribution defined in terms of its mean and standard deviation [38]. A wide variety of prior probability distributions are available [65]. For this study a normal probability distribution was used as it is thought to better reflect uncertainty related to secondary calibration points [65]. For dataset A, a mean of 91 and a standard deviation of 1.5 were specified. This effectively encloses a range of possible ages from c. 89 to 93 Myr. In dataset B, a normal distribution was also used for the age of the stem node of *Isolona *and *Monodora*, with the mean taken from the analysis of dataset A. The standard deviation was set to contain the lower and higher boundaries of the 95% highest posterior density values effectively accommodating for age uncertainty.

Both sequence datasets (A and B) deviated from a strict molecular clock and rates between adjacent branches were uncorrelated as shown by the values of the parameters 'coefficient of variation' and 'covariance', respectively [66] (Table 1). Thus divergence times were estimated under a lognormal non-correlated relaxed clock method and using the Yule model of speciation as implemented in BEAST 1.4.7. In all cases user-specified chronogram trees were used as starting trees and were obtained using the r8s program [67]. Finally, taxon subsets were specified for each clade of interest allowing recording of the mean time of the most recent common ancestor (*t*_MRCA_), the 95% highest posterior density intervals (HPD), and the effective sampling size (ESS). Dates were considered significantly different only when the 95% HPDs were not overlapping.

**Table 1:** Mean and 95% of the highest posterior distributions (HPD) of the coefficient of variation and covariance parameters for dataset A (genus-level phylogeny) and B (species-level phylogeny)

	Coefficient of variation	Covariance
**Mean dataset A **(30 million generations, sampled every 1000th)	**0.525**	**7.33E-02**
95% HPD lower	0.45	-0.101
95% HPD upper	0.602	0.238
**Mean dataset B **(5 million generations, sampled every 1000th)	**0.753**	**-4.52E-02**
95% HPD lower	0.521	-0.254
95% HPD upper	1.012	0.186

## Results

### Divergence times

**Figure 2:**
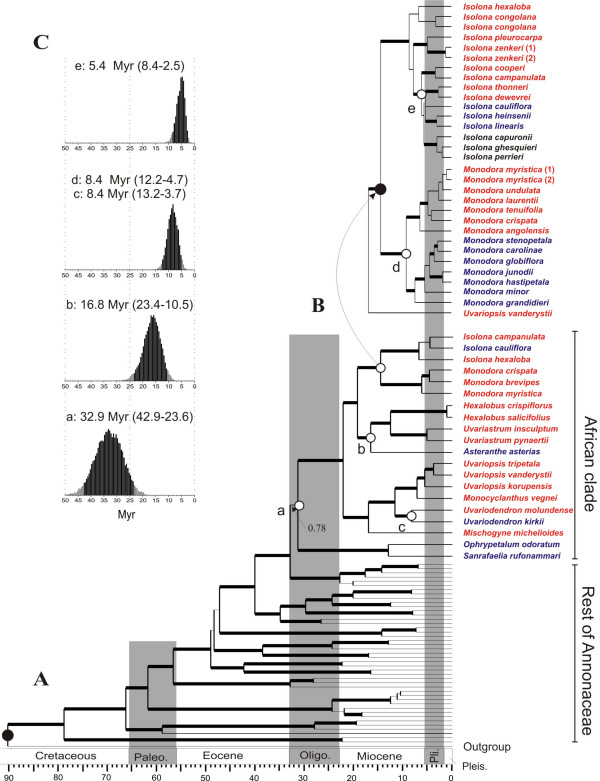
**Estimated divergence times within African Annonaceae**. Maximum clade credibility chronograms, with nodes represented by their mean ages estimated under a relaxed lognormal uncorrelated molecular clock assumption. East African endemic taxa are indicated in blue, West and Central African taxa in red, taxa endemic to Madagascar in black. Solid circles indicate nodes used for calibration of the trees. Open circles indicate nodes for which divergence dates were estimated. Thick branches lead to nodes with more than 0.95 posterior probability support. Geological Epochs, shaded bars: Paleo., Paleocene; Oligo., Oligocene; Pli., Pliocene; Pleis., Pleistocene. (A) Genus-level chronogram showing phylogenetic relationships within the African clade. (B) Species-level chronogram of the two sister genera *Isolona *and *Monodora*. (C) posterior distributions of the estimated ages. The 95% highest posterior density (HPD) intervals are indicated with black bars and given between brackets after the mean. These distributions were used to accept or reject significant congruence of node ages.

Our results provide strong support for the multiple connection-reconnection hypothesis with relative ages of splits occurring at three significantly different periods in time (Figure 2). The estimated absolute ages for all of the major clades recognized within Annonaceae are in perfect agreement with recent studies [24,50]. Within the African clade five different origins of endemic East African lineages were identified (Figure 2A and 2B, nodes a-e). Node 'a' represents the origin of the *Sanrafaelia*/*Ophrypetalum *clade with a mean age of 32.9 Myr (95% HPD: 42.9–23.6 Myr) and node 'b' that of *Asteranthe *with a mean age of 16.8 (HPD: 23.4–10.5). Node 'c' indicates the origin of the East African endemic species *Uvariodendron kirkii*, with a mean of 8.4 Myr (HPD: 13.2–3.7). Two further nodes are indicated in Figure 2B, with the first one corresponding to the origin of the East African species of *Monodora *(node 'd'), with a mean of 8.4 Myr (HPD: 12.2–4.7). The lack of phylogenetic resolution makes it impossible to distinguish the origin of the East African species of *Isolona *(Figure 2B). Therefore, only a minimum age for this potential split can be provided corresponding to node 'e' with a mean of 5.4 Myr (HPD: 8.4–2.5). Each age estimate (Figure 2C) for these nodes is dated to three significantly different periods of time as the 95% HPDs do not overlap. For node 'c', 'd', and 'e' the 95% HPDs do largely overlap, indicating no significant differences in the age estimates.

### Phylogenetic analyses

The chronograms and phylogenetic relationships resulting from both analyses are presented in Figure 2A and 2B. At the generic level (dataset A) the resulting phylogenetic relationships as well as corresponding branch support were identical to those found in Couvreur et al. [18]. The species-level chronogram (dataset B) of the two sister genera *Isolona *and *Monodora *is represented in Figure 2B. *Monodora *has two well-supported sister clades (Bayesian posterior probability – *PP *= 1.00): a West African clade, and an East African clade. Relationships within the West African clade are weakly supported except for the sister position of *Monodora angolensis *(*PP *= 1.00) to the remaining West African species. In the East African clade, *Monodora grandidieri *is strongly supported as sister to the rest of the taxa. Moreover, *Monodora globiflora *with *Monodora carolinae *and *Monodora stenopetala*, as well as *Monodora hastipetala *with *Monodora junodii *form well-supported subclades (*PP *= 1.00). The Malagasy species of *Isolona *form a highly supported, nested clade. The East African taxa are also strongly supported as nested within *Isolona*; however, their relationship with the other clades remains unresolved.

## Discussion

Our results demonstrate that the splits between the Guineo-Congolian and East African Annonaceae lineages have multiple origins at significantly different times (Figure 2), which is congruent with the hypothesis presented in Figure 1C. Given that the majority of species found within this clade are restricted to rain forests and present no long-range dispersal potential, we interpret the observed pattern as a likely consequence of a series of connection-isolation events between the East African and Guineo-Congolian forests. Our results were obtained with just one plant group, but the biogeographic patterns observed in Annonaceae are common amongst many other tree families in Africa in terms of ecology (taxa restricted to rain forests) and distribution (disjunct between West/Central and East Africa) [8,11,12]. Therefore, we predict that the evolutionary process of divergence by vicariance on multiple occasions could be a common pattern for numerous other plant taxa in Africa, thus explaining much of the plant distribution and endemicity within African rain forest tree families. Interestingly, multiple splits between East and West-Central rain forest dwelling lineages at different times were also found to have occurred in African caecilian amphibians [48]. Thus, it would appear that this process could also be the main factor behind present day distribution patterns for a wide range of African rain forest restricted organisms, including animals.

Our results also provide important insights into the absolute time scale of these events, especially for the origin of the East African endemic lineages. The dates inferred from our study were obtained based on time calibration using the fossil taxon *Archaeanthus *(see Methods) as it is generally accepted to represent a minimum age constraint for the stem of Magnoliaceae [24,49,50,53,58,59]. Even acknowledging the complex history of vegetation change in East Africa [16] and the uncertainty in age estimates, the estimated origins of these splits using the *Archaeanthus *fossil calibration coincide well with known periods of renewed aridity and/or continental uplift in East Africa. These phenomena are generally thought to have played a role in the break-up of the pan-African rain forest, thus providing an explanation for the observed splits, which can be looked upon as further support of their correctness. The first split is dated to the Oligocene (mean: 32.9 Myr; Figure 2A – a), which corresponds to a period of drastic global cooling resulting, in part, from the development of permanent continental ice-sheets in Antarctica [68]. This cooling induced a drier climate at equatorial levels in Africa, fragmenting the pan-African Eocene rain forest [14]. The second split, dated to the Late-Early Miocene (mean: 16.8, Figure 2A – b), post-dates an Early Miocene warmer and moister climate during which rain forest is thought to have extended again from coast to coast [13,15], and coincides with the Miocene climatic optimum (17-15 Myr) characterized by high global temperatures [68]. This period also witnessed the closure of the Tethys sea when the African plate collided with the Eurasian one (c. 18-17 Myr), bringing an end to the moist influence of the latitudinal oceanic circulation system [15,69]. These drier conditions, as well as higher global temperatures, induced a new period of marked aridity, allowing the spread of savannas at the expense of rain forests [15,69]. This is visible at the paleobotanical level by an increase in grass abundance across Africa by 16 Myr [16]. The third significant split (mean: 8.4–5.4 Myr) occurred in three lineages, *Uvariodendron *(Figure 2A – c), *Monodora *(Figure 2B – d), and *Isolona *(Figure 2B – e), and took place after the initiation of geological activity in the western East African Rift System that uplifted the central Tanganyikan plateau (c. 10 Myr) [12,70]. Such uplifting has been shown to have played a significant role in the aridification of eastern Africa [70]. Moreover, this estimated date also coincides with a period of significant extension of savannas in East Africa [16,69], as judged by an increase in biomass of plants using C_4 _photosynthetic pathways (8-6 Myr) [71].

Furthermore, even with our fossil-based minimum age estimates most speciation events in East African *Isolona *and *Monodora *(Figure 2B) have a pre-Pleistocene origin indicating a lack of diversification in the East African rain forests during the climatic fluctuations of the Pleistocene (e.g. [72]). For East Africa, this pattern was also documented in clawed frogs where all extant East African taxa were found to have originated before the Pleistocene [73]. Paleoecological records from the Eastern Arcs in Tanzania spanning the last 38,000 years provided evidence of ecosystem stability during the last glacial maximum period [74]. This lack of diversification during the Pleistocene is generally thought to be due to the stabilizing moist influence of the Indian Ocean on tropical rain forests in parts of coastal and montane East Africa, allowing them to persist throughout glacial periods without any important fluctuations in size [12,75]. Therefore, our results provide strong evidence that a larger Oligocene-Miocene continental-scale fragmentation between, rather than within, East and West-Central rain forest regions has played a major role in generating the patterns in plant distribution and endemicity that are currently observed across Africa. However, it may be that other, possibly herbaceous, groups diversified in East Africa as a result of Pleistocene climatic change but this is certainly not the case for Annonaceae and is likely also not true for other woody representatives of the East African rain forest flora.

Finally, our results indicate that the highly fragmented and threatened rain forests of Eastern Africa contain large amounts of pre-Pleistocene derived endemic lineages having evolutionary histories that date back to the Early Oligocene. A large majority of these lineages are species-poor [11], making them vulnerable to extinction. Additionally, most of the endemic species or genera are highly vulnerable to extinction because of small distributional ranges [9]. Subsequently, they have been red-listed with some level of threat to their survival (an estimated 22–25% of the total number of endemic species [Roy Gereau, personal communication]). Thus, because of their ancient origins and species-poor composition, the extinction of just a handful of species could result in the loss of significant phylogenetic diversity [6]. This information should be carefully and urgently taken into consideration in future conservation planning within the East African region as knowledge of the presence of such ancient lineages allows us to prioritize areas with high phylogenetic diversity [6,76,77].

## Conclusion

Our molecular phylogenetic study on the evolutionary history of this pan-African Annonaceae rain forest clade provided numerous important insights into the evolutionary origins of African rain forest trees. We show that the East African lineages within this large clade have significantly different temporal origins. This would suggest that present day distribution and endemicity in African rain forest is a likely consequence of a series of connection-isolation events between the East African and Guineo-Congolian forests. Moreover, all the East African taxa appear to have diversified before the Pleistocene climatic oscillations. This provides evidence for the increasingly probable hypothesis that East African rain forests have been ecologically stable during those times. Finally, the old pre-Pleistocene origins of the endemic East African taxa suggest that these biodiversity hotspot regions contain high levels of phylogenetic diversity, an important factor to take into account for conservation priorities.

## Abbreviations

Myr: million years; *PP*: Bayesian posterior probability; HPD: highest posterior distributions.

## Authors' contributions

TLPC carried out the molecular genetic studies, the sequence alignment, phylogenetic and dating analysis, and drafted the manuscript. LWC participated in the sequence alignment, supervised the study and helped to draft the manuscript. MSM participated in the design of the study. JER conceived the study, and participated in its design and coordination, and helped to draft the manuscript. All authors critically read and improved earlier drafts and approved the final manuscript.

## Supplementary Material

Additional file 1 **Vouchers and genebank info**. Taxon sampling, voucher information and GenBank accession numbers for each of the six (dataset A) and five (dataset B) chloroplast markers used in this study. UUCB: University Utrecht Botanical Garden; DRC: Democratic Republic of Congo. Click here for file
